# Implementing integrated community case management during conflict in Yemen

**DOI:** 10.7189/jogh.10.020601

**Published:** 2020-12

**Authors:** Nathan P Miller, Nureyan Zunong, Taha Ali Abdulrahman Al-Sorouri, Yasmin Mohammed Alqadasi, Sarah Ashraf, Cashington Siameja

**Affiliations:** 1 UNICEF, New York, New York, USA; 2 Mailman School of Public Health, Columbia University, New York, New York, USA; 3 Save the Children US, Washington, D.C., USA; 4 Yemen Ministry of Public Health and Population, Sana’a, Yemen; 5 KIT Royal Tropical Institute, Amsterdam, Netherlands; 6 Save the Children Yemen, Sana’a, Yemen

## Abstract

**Background:**

The conflict in Yemen has devastated the health system, with only 51% of health facilities classified as fully functional and 19.7 million people lacking access to health care. To address the urgent need for primary health care services in rural communities, Save the Children launched an iCCM program in Lahj and Taiz Governorates. A qualitative study was conducted to document the challenges to iCCM service delivery and to aid in developing strategies for overcoming service delivery bottlenecks in conflict-affected rural areas.

**Methods:**

Qualitative data were collected in Aden City, Lahj Governorate, and Taiz Governorate. Twenty-three IDIs and six FGDs were conducted with iCCM stakeholders at all levels.

**Results:**

Key findings included: 1) Policy, coordination, and funding were challenged by the fact that iCCM was not integrated into the national health system and was implemented as a short-term emergency program. 2) Villages that received services from a CHW who was based in a different community experienced reduced access to services, especially during times of heightened conflict and insecurity, when CHWs could not travel. 3) Supervision, supply chain, and monitoring were all challenges that were exacerbated by difficulties in travel due to the conflict. Potential solutions to these included the use of mobile technology for supervision and data collection and pre-positioning of buffer stocks in locations closer to CHWs. 4) Travel was seen as the primary threat to the safety of CHWs and supervisors. Measures taken to reduce the risk included limiting travel during periods of heightened insecurity, safety training for CHWs, and use of mobile technology for communication.

**Conclusions:**

CHWs were able to provide iCCM services in a challenging and insecure context. The challenges in delivery of services were related to both a weak health system and the conflict. Several adaptations to service delivery to overcome the bottlenecks have been identified and should be considered for future community health programs. The closure of the program in Taiz after only 14 months of implementation is a stark illustration of the failure of the current model of short-term humanitarian funding to address long-term needs in protracted emergencies.

Integrated community case management (iCCM) of childhood illness is a strategy to train, equip, and support community health workers (CHWs) to diagnose and treat childhood pneumonia, diarrhea, and malaria at the community level [1]. Studies have shown that CHWs can provide relatively high quality care [2-6] and that community management of common childhood infections (eg, diarrhea, pneumonia, malaria) can substantially reduce child mortality [7-11]. Given the high burden of these illnesses in humanitarian settings, there is a need to assess the feasibility of implementing iCCM in these complex environments. Studies on iCCM and other community-based maternal, newborn, and child health services in emergency settings have shown that CHWs can continue providing routine services and can improve emergency response if they are adequately supported [12-17].

The escalation of conflict in Yemen in 2015 exacerbated an already existing protracted crisis. As of December 2018, an estimated 80% of the population (24.1 million people, including 7.4 million children) is in need of humanitarian assistance and 4.3 million people had been displaced in the previous three years. The conflict has devastated the health system, with only 51% of health facilities classified as fully functional and 19.7 million people lacking access to adequate health care [18].

To address the urgent need for primary health care services in rural Yemeni communities, Save the Children International (SCI) launched an iCCM program in Lahj and Taiz Governorates in July 2017. SCI recruited 40 CHWs in Lahj and 40 in Taiz. The CHWs were trained to provide treatment to children under five years of age for uncomplicated pneumonia, diarrhea, and malaria, and to identify severe cases for referral. CHWs also carried out education on health, nutrition, and hygiene, as well as early detection and referral of children with acute malnutrition to nutrition services. The project aimed to reach a total of 35 785 children under five years of age. CHWs were mostly women and were paid US$100 per month in Lahj and US$80 per month in Taiz. All CHWs had at least secondary education and some had some medical background. The project was closed in Taiz in September 2018 and the project is ongoing in Lahj.

SCI, with support from UNICEF and the Yemen Ministry of Public Health and Planning (MoPHP), conducted a qualitative study to document the challenges to iCCM service delivery and to develop strategies for overcoming service delivery bottlenecks in conflict-affected areas of Yemen.

## METHODS

### Study sites

**Figure 1:**
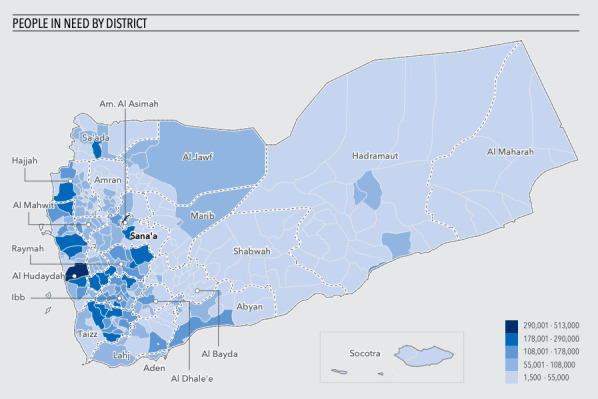
Map of people in need by district in Yemen. Source: UNOCHA [18].

Data were collected in Aden City, Lahj Governorate, and Taiz Governorate (**Figure 1**). National stakeholders were interviewed in Aden City. Data for Lahj were collected in Al-Hawta City and Toban District. Taiz data were collected in Taiz City, Al-Torba City, Al-Nashma City, Ibb City, Al-Misrakh District, and Al-Maafer District.

Lahj and Taiz were affected by the conflict directly, with bombings, conflict, and road closures, and indirectly, with deterioration in economic activity, food shortages, and health system failures. Many health facilities were closed and either partially or completely destroyed, and there was a severe lack of human resources for health.

### Study respondents

Informant groups included: 1) policy makers who were knowledgeable about the country context, the health system, and specifically about iCCM in the country, such as MoPHP officials, United Nations (UN) staff, donors, and non-governmental organization (NGO) representatives, 2) program implementers, including MoPHP officials and NGO iCCM coordinators, program managers and officers, 3) health facility staff, 4) CHW supervisors, 5) CHWs delivering iCCM, 6) community leaders, and 7) caregivers of children under five years of age.

### Data collection tools

The study instruments were developed by SCI and UNICEF, with input from the MoPHP. The tools were adapted from standardized tools developed by UNICEF for a series of studies on CHWs in humanitarian settings, including studies in Guinea, Liberia, and Sierra Leone during Ebola [13], South Sudan during conflict [12], and Bangladesh during flooding [14]. Separate data collection tools were developed for different categories of respondents and for each data collection method.

### Data collection procedures

Data were collected in December 2018 and January 2019, about a year and a half after the start of implementation of the iCCM program. One data collection team, consisting of two experienced Yemeni researchers, carried out all data collection.

Semi-structured in-depth interviews (IDIs) were conducted with policy makers at the national level, program implementers at the national, governorate, and district level, health workers in referral facilities, CHW supervisors, and community leaders.

Focus group discussions (FGDs) were conducted with CHWs providing iCCM services and caregivers of children under five years of age. Each FGD group consisted of six to nine participants.

### Sampling and sample size

Study participants were selected using purposive, non-probability sampling. Investigators selected the policy makers and iCCM program implementers at the national level through discussions with the MoPHP focal point and the SCI country office. At the governorate and district levels, program implementers were selected from both SCI and the MoPHP. Health workers who provided referral care for children referred from CHWs were also included. The study team worked with SCI and the MoPHP to identify the CHWs and CHW supervisors working in a district. CHWs were asked to assemble at a health facility, district administration office, or other central location so they could participate in FGDs. For data collection with community members, the research team traveled to communities and interviewed community leaders with knowledge of the community’s experience of the emergency and of iCCM services. They also engaged local guides, community leaders, and CHWs to assist in selecting caregivers of children to participate in FGDs.

**Table 1** shows the final sample size by type of respondent and method of data collection. In total, 23 IDIs and 6 FGDs were conducted. The number of respondents was nearly identical between Lahj and Taiz Governorates, with the only difference being one additional community leader interviewed in Taiz.

**Table 1:** Respondents, methods, level of data collection, and numbers of IDIs and FGDs

Respondent	Method	Level	Number
Policy makers	IDI	National	2
Policy makers and program implementers	IDI	Governorate	8
Policy makers and program implementers	IDI	District	2
Health workers	IDI	District	4
CHW supervisors	IDI	District	4
Community leaders	IDI	Community	3
CHWs	FGD	District	2
Caregivers	FGD	Community	4
**Total**			**29**

FGD – focus group discussion, IDI – in-depth interview

### Data management and analysis

IDIs and FGDs were audio recorded and transcribed verbatim in Arabic. The transcriptions were then translated into English for analysis. Analysis of English transcripts was performed by a separate researcher. The data were coded by hand and themes were identified using a combination of deductive and inductive thematic analysis. The deductive framework was based on the components of the iCCM benchmarks framework [19]. Additional themes and sub-themes were determined inductively based on the information that appeared in the data.

### Ethical concerns

Ethical approval was obtained from the Yemen MoPHP. Informed consent was obtained from all study participants prior to data collection.

## RESULTS

### Policy

Given that iCCM was not integrated into the national health system and there was no existing policy, strategy, action plan, or guideline for implementing iCCM, several respondents suggested that integrating iCCM into the national health system would improve service delivery and sustainability of services. They advocated for a national iCCM policy, strategy, action plan, guideline, and standard criteria for selecting and remunerating CHWs.

Several people also argued for a number of additional interventions to be added to the package of services provided by CHWs. These included maternal and newborn health, immunization, reproductive health, and nutrition services. Several respondents argued that CHWs should be more systematically trained and involved in control of epidemic-prone diseases, such as cholera and dengue. An implementer discussed the role of CHWs during the cholera outbreak: “*We have a successful experience in that when the cholera epidemic spread in Taiz ...CHWs had a major role in educating people and preventing cholera through distributing ORS* [oral rehydration salts], *educating people and mothers in the way to prepare ORS at home and how to use it properly. Actually this was one of the success stories of iCCM in Taiz, which must be marketed and disseminated to all stakeholders, particularly the health sector, donors, and international organizations.*” Finally, several respondents advocated for CHWs to be trained to provide first aid.

### Funding

Policy makers and implementers lamented a dearth of funding for the health sector in general and for iCCM in particular. More funding was needed to train CHWs, to expand the geographical coverage of iCCM, and to fund a functional referral system. However, instead of expanding services, the project in Taiz has now ended due to a lack of funding. With very short-term donor funding and no funding for iCCM from the government budget, the project was not sustainable. To improve the funding situation for iCCM, respondents suggested advocating to donors about the importance of iCCM and coordinating funding among donors and including iCCM in the UN emergency response plan.

Respondents suggested integrating iCCM into the national health system to improve the funding and sustainability of iCCM services, as discussed above. If iCCM becomes part of the national health system and is included in the five-year government health plans, it will be much easier to advocate for funding from donors. As an implementer in Taiz explained, “*A guideline for iCCM should be prepared ... Availability of this guideline with all details will improve the funding, as the donors are obliged to meet the requirements of the MoPHP. Generally, the donor will accept to fund a project if its services will be implemented according to the MoPHP guideline.*”

### Coordination

Coordination was complicated by the conflict. As an implementer in Taiz explained, “*Most stakeholders in the DHO* [district health office] *of the targeted districts were out of the district because they had been displaced to other areas. So we faced challenges in reaching them, coordinating and arranging with them for iCCM implementation.*” As with policy, respondents advocated for better coordination with the government at all levels and for the government to eventually take the lead in coordinating iCCM activities.

### Recruitment of CHWs

The initial criteria for recruitment of CHWs was that they have a medical background. It proved difficult to find a sufficient number of candidates with that background, so the requirement was changed. An implementer in Lahj explained, “*If we need two CHWs, we only found one CHW who met the criteria because in the same village there are few educated people who meet the required qualification.*“ It was especially difficult to find women, who were prioritized, who had a medical background. In response to this, the criteria were changed from medical background to secondary education. It was still difficult to find a qualified candidate in every village, so in some cases CHWs were recruited from neighboring villages.

### Coverage of and access to iCCM services

CHWs were not evenly distributed among villages. A supervisor in Taiz explained the situation, saying, “In some villages we faced difficulties in finding CHWs who meet the criteria while in other villages we could find a lot of CHWs who meet the criteria, so this leads to inequity in distribution of CHWs.”

As some CHWs covered multiple villages, those villages that were more remote may have had lower access to care. The CHWs complained that they incurred high transportation costs to travel to these remote villages, which they were not reimbursed for. Furthermore, during the rainy season the roads of some areas were blocked due to floods.

In the case of increased conflict and insecurity, villages where the CHW is not a resident, and particularly remote villages, may see a drastic reduction in access to iCCM services. As a health worker in Taiz explained, “*If there is escalation of conflict, CHWs will provide services only in their village, not in other villages, because it will be difficult for them to move to other villages in such a context.*”

Respondents confirmed that displaced persons in areas covered by the iCCM program were benefiting from the services. A community leader in Taiz said, “*There is a camp for internally displaced people [IDPs] in this area, specifically in the tier two catchment area of Faiuosh health center. IDPs are able to receive CHWs’ services because they reside within the catchment area that the CHWs should serve. CHWs visit the IDP camp regularly to deliver services*.” However, the influx of IDPs in some areas had strained the CHWs because, although the catchment population had increased, the number of CHWs to serve them had not.

In one area in Taiz that had experienced conflict, caregivers confirmed that, “*She* [the CHW] *is willing to continue providing services. She hasn’t moved during days of war.*” Caregivers in Lahj also said that their CHW would move with them and continue to provide services if they were displaced.

A number of suggestions were put forward by respondents to resolve some of the challenges around coverage and access. First, iCCM services need to be expanded to additional areas without access to health care. To improve access to services in hard-to-reach areas, CHWs should be given a transport allowance to cover the cost of travel to remote villages. To ensure adequate access to CHWs in all villages, caregivers suggested that, “*CHWs should reside in the same village they serve or in nearby village that facilitate contacting and communicating with them.*”

### Supervision

Implementers from SCI cited distance and difficult terrain to many villages as factors limiting regular supervision. Government staff either did not have sufficient time or knowledge to effectively supervise CHWs. An implementer in Taiz explained, “*Because of the situation in Taiz, in addition to* [the fact] *that government health staff is without salaries and many of them have moved to work in the private sector, we have not been able to appoint direct field supervisors from the GHO* [governorate health office]. *In addition, we have not been able to involve DHOs* [district health office] *because most health officers have no idea about iCCM ...*”

There were additional challenges caused by the conflict and insecurity. A supervisor in Lahj said, “*Sometimes explosions and confrontations or clashes prevent supervisors from making field visits, so visits were postponed ...*” There were also difficulties to supervise some areas when roads had been flooded during the rainy season.

When it was not possible to reach some areas to provide direct supervision, CHWs were contacted by mobile phone or email. A supervisor in Taiz explained, “*We have had difficulty in reaching some areas, especially in areas where there is armed conflict. Also, sometimes there are security problems and we receive instructions...not to go to the field. In order to deal with this, we were communicating with CHWs through WhatsApp.*” CHWs in Taiz also created a WhatsApp group to discuss problems, exchange ideas, and give each other feedback.

Suggestions for improving supervision included increasing the number of supervisors, having health facility workers provide supervision, and providing CHWs with telephones so they can more easily contact supervisors, health facility workers, community members, and each other. One respondent also proposed selecting supervisors from the same communities as CHWs so they can more easily reach CHWs for supervision and using peer supervision.

### Supply chain

Shortages of iCCM drugs were cited as a primary limitation of the program by nearly all respondents. There were several reasons for this. First, the amount of drugs budgeted for in the project proposal was not sufficient. Second, when drugs were ordered from international sources, there were delays in receiving them into the country. Third, in some cases, drugs that were provided to health facilities to supply CHWs were consumed in the health facilities before they could reach the CHWs.

Conflict and insecurity played a large role in causing drug stockouts. At times, the ports closed, so it was impossible to import medicines. Supplies from Sana’a to Lahj needed to pass through conflict-affected areas, which caused supply chain delays during times of heightened conflict. A supervisor described a period when supplies could not be received in Taiz: “*The supply chain ... was interrupted because Taiz was besieged at the time. Even though the Taiz-Altorbah-Aden road was open, the GHO could not provide us with supply because the GHO’s stocks were exposed to explosion and burning.*”

The main suggestion for improvement of drug supplies, given by implementers and supervisors in Lahj and Taiz, was to provide buffer stocks, either in health facilities or near communities. As a supervisor in Taiz said, “*Buffer stock should be provided for the CHWs ... So that if there is any problem in the health facility, CHWs have a quantity sufficient for two months. Or a buffer stock for a group of CHWs in a suitable location, such as a central area that is accessible for CHWs. This stock can be used if there is a problem in the supply due to conflict or insecurity.*”

### Service utilization and demand generation

In addition to a general lack of trust in the health system, some communities were reluctant to accept CHWs without medical qualifications. Drug stockouts also caused a loss of confidence in CHWs among community members. Demand generation efforts were carried out to encourage communities to utilize iCCM services. An implementer in Lahj explained, “*To promote community acceptance of CHWs, a mitigation plan was developed. We conducted meetings with community members, community leaders, and village heads to clarify the importance of CHW services, what their skills were ... These procedures were fruitful and effective.*” By the end of the project, all respondents reported high levels of utilization and good relationships between CHWs and community members.

The conflict was reported to have both negative and positive effects on iCCM service utilization. A supervisor in Lahj explained, “*We have faced difficulties in Aden that people do not open the door to CHWs when they knock because of reasons related with rumors about terrorism and house attack.*” Social mobilization to promote service utilization was also made difficult in insecure areas. On the other hand, increased insecurity was seen as promoting utilization of CHW services in some cases. According to an implementer in Lahj, “*Conflict resulted in a lack of service packages of health facilities. One of the iCCM project’s advantages is that CHWs are providing services during conflict in a time when it is difficult for people to go to the health facility for security reasons ... Many of the health facilities were either destroyed or looted ... so the most appropriate way to provide health services in these cases was iCCM.*” A caregiver in Lahj added, “*During heightened conflict, utilization of CHW services will increase. When my child becomes ill, I search for any possible treatment, especially when we are unable to go to a hospital or clinic.*” Respondents also reported that areas that received displaced persons saw increases in utilization, which led to drug shortages.

### Monitoring and evaluation

Monitoring and evaluation of iCCM services faced many of the same challenges as supervision. The primary means of overcoming these challenges was to use mobile phones to transmit data. An implementer in Taiz explained, “*Because most targeted villages are remote areas, we could not collect the CHWs’ reports weekly. So we used WhatsApp for receiving CHWs’ reports and providing feedback. Then during monitoring field visits the supervisors collected the reports.*”

### Referral

The primary challenge for patients referred by CHWs to health facilities was the cost of transportation. Respondents affirmed that many patients in remote rural areas were not able to afford the cost of transportation to a health facility and therefore did not complete referral. Respondents also complained that there was no integrated referral system that linked CHWs to health facilities. For example, there were no referral forms to be sent with patients and there was no feedback from health facilities on whether referred patients arrived and were managed at the health facility. There were also some challenges with health facilities’ ability or willingness to manager ferred patients. Some health facilities refused referral patients because they were not involved in selecting CHWs. Furthermore, some referral facilities were not well equipped to treat children as they lacked the necessary drugs and equipment.

### Safety of CHWs and supervisors

Although there were no reported incidents of CHWs experiencing direct threats to their safety, insecurity and the conflict did affect their work. A health worker in Lahj affirmed, “*Whenever there is an escalation of conflict or insecurity in the region, the safety of CHWs and their supervisors is at risk because there will be difficulties in movement and supply, especially if the CHWs provide services in remote villages other than their own.*” Several respondents described moments of increased insecurity in some areas that disrupted CHWs’ and supervisors’ work. An implementer in Taiz said, “*In general, we did not face challenges in the safety of CHWs and their supervisors, but the CHWs in the villages located in the confrontation line have experienced great difficulties in their safety and the safety of their supervisors.*” It was felt that the primary risk would be for CHWs who covered multiple villages and therefore had to travel and for supervisors who had to travel to several villages for supervision. A policy maker in Lahj said, “*If an escalation occurs...the safety of their supervisors will be at risk as they have to move to reach to CHWs and the road will not be safe.*”

Both SCI and communities took actions to minimize the risk to CHWs and supervisors. SCI conducted meetings with community leaders to ensure that community leaders took the responsibility to ensure the safety of CHWs and to facilitate their work. CHWs were encouraged to exercise caution in their work. For example, a health worker in Lahj explained, “*We delay any activity of the CHWs if there is any danger in the targeted area. Furthermore, if we face a security threat, we stop the project’s activities temporarily until the threat ends. What is important for us is the safety of CHWs and not to endanger them.*” A supervisor in Taiz explained their approach to CHW and supervisor safety: “*Some of the targeted areas are located in the line of confrontation between parties to the conflict, in which there were clashes and bombing ... Therefore, we supported CHWs with awareness on safety issues for them and the community in such a context. Also we gave them instruction to hold awareness sessions for the community only in safe times. Of course, we, the supervisors, could not reach these areas because the organization’s security* [policy] *prevented us* [from traveling] *for security reasons. We have been communicating with CHWs through WhatsApp.*” To avoid threats to female CHWs who have to travel to other villages, male and female CHWs were grouped together to travel to remote areas.

Respondents suggested several measures to ensure the safety of CHWs during times of increased insecurity. These included having CHWs serve only in their home villages, coordinating with community leaders to ensure CHWs’ safety, and providing security training to CHWs.

## DISCUSSION

The study showed that the conflict and related insecurity did complicate iCCM service delivery. However, many of the service delivery challenges were due to factors more related to the general weakness of the health system than to the conflict. This is similar to findings from the Ebola context that showed that the strength of service delivery prior to the emergency (whether through the government health system or supported by implementing partners) was a strong determinant of whether services would be maintained during the outbreak [13].

The importance of integrating community health services into national health systems, which has been advocated for previously [20], was highlighted again in this study. The lack of ownership of iCCM by national and district health officials and health facility staff hampered both service delivery and sustainability. These findings also add to previous studies that emphasized the importance of longer-term, sustainable funding with the flexibility to transition between development and humanitarian settings [12,21].

The importance of recruiting CHWs to work in their home communities was emphasized for several reasons. Having local CHWs increased availability of services and improved community members’ trust in the CHW. It was also felt that in times of increased insecurity, CHWs would not be able to travel to distant villages. If CHWs are expected to travel outside of their home communities, costs for reimbursement for travel expenses should budgeted for. The need for a CHW in each village will have implications on the number of CHWs who need to be recruited and on the level of education that CHWs will have. Recruitment criteria need to be realistic given the education level of people in rural communities. The challenge of recruiting literate CHWs, especially in the case of female CHWs, has been shown in other contexts, such as the Democratic Republic of Congo, Liberia, Sierra Leone, [22] and South Sudan [12].

As in South Sudan [12], population displacement led to an increased burden on CHWs in communities that received IDPs, as well as shortages of drugs and supplies due to the increased population. In contexts with high population displacement, programs should be flexible in terms of being prepared to quickly deploy additional CHWs and increased levels of commodities and supplies in areas that receive IDPs.

Supervision of CHWs was inconsistent and often did not include assessment of performance due to a lack of expertise on the part of supervisors. Having supervisors as close to the communities as possible would also help ensure continued supervision and monitoring during times of increased insecurity. The study on iCCM during conflict in South Sudan also showed that having supervisors from the local communities aided their ability to use local networks to assess the security situation and to track down displaced CHWs [12]. The use of mobile technology such as WhatsApp was also useful in allowing continued contact between supervisors and CHWs and among CHWs during times when travel was difficult.

Stockouts of iCCM commodities were a major hindrance to effective service delivery. If drugs pass through health facilities to CHWs, it is necessary to ensure sufficient drugs are available at health facilities so they don’t consume the drugs intended for CHWs. Therefore, an effective CHW program may also require support to referral health facilities. To avoid stockouts due to the conflict, it may be necessary to locate storage facilities closer to communities or to provide CHWs with larger buffer stocks so that they will have continued supplies when routine distribution is disrupted. These measures were seen as beneficial in several other studies of CHWs in emergencies [12,15,23,24]

Some communities were reluctant to utilize CHW services, which indicates the need for strong communication and social mobilization efforts when iCCM services are introduced. The importance of strong community mobilization in achieving impact of iCCM has been shown previously in Ethiopia [6]. These efforts will be most effective if community leaders are convinced of the value of CHW services and are engaged in community mobilization, which was also shown in the Ebola context [13].

Referral was a challenge in non-emergency settings that was further exacerbated by conflict and insecurity. As in Afghanistan [23], the primary difficulty for caregivers to complete referral was the cost of transportation. A large number of respondents stated that caregivers could not afford to take a referred child to a health facility. Therefore, some form of facilitated referral or subsidization of transportation costs would be necessary for caregivers to consistently complete referrals.

Finally, although the majority of respondents stated that CHWs and supervisors had not faced direct security challenges due to the conflict, it was clear that their safety could be threatened during times of increased insecurity. This is especially true for supervisors, who have to travel to communities, and for CHWs who cover multiple villages. During times of heightened insecurity, movement will be limited. Therefore, having CHWs who work only in their own communities will increase the safety of CHWs. For supervision, monitoring, and supply chain, the measures discussed above, such as using mobile technology to supervise and receive reports and providing buffer stocks to CHWs, will help reduce security threats.

This study has several limitations. First, the impact of the conflict in Yemen varied substantially by region, so the results of an iCCM program in other regions with a greater or lesser level of conflict and insecurity may have been different. Second, we were not able to obtain quantitative iCCM service delivery data that would have allowed us to triangulate the qualitative data. Third, the sample size was limited by the available budget, so we may not have reached the point of saturation. Finally, community-level respondents who were selected by CHWs may have been biased in favor of the CHWs.

## CONCLUSIONS

CHWs were able to provide iCCM services in a challenging and insecure context. The service delivery challenges were related to both a weak health system and the conflict. Adaptations to service delivery to overcome bottlenecks, such as use of mobile technology and adapting supply chain and security procedures, should be considered for future community health programs. The closure of the program in Taiz after only 14 months of implementation is a stark illustration of the failure of the current model of short-term humanitarian funding to address long-term needs in protracted emergencies.
